# Revisiting Hemispheric Asymmetry in Mood Regulation: Implications for rTMS for Major Depressive Disorder

**DOI:** 10.3390/brainsci12010112

**Published:** 2022-01-14

**Authors:** Benjamin C. Gibson, Andrei Vakhtin, Vincent P. Clark, Christopher C. Abbott, Davin K. Quinn

**Affiliations:** 1 Psychology Clinical Neuroscience Center, University of New Mexico Psychology Department, Logan Hall, MSC03-2220, 1 University of New Mexico, Albuquerque, NM 87131, USA; bcgibson@unm.edu; 2 The Mind Research Network/Lovelace Biomedical and Environmental Research Institute, 1101 Yale Blvd. NE, Albuquerque, NM 87106, USA; avakhtin@mrn.org; 3 Department of Psychiatry and Behavioral Sciences, University of New Mexico School of Medicine, 2600 Marble Avenue NE, Albuquerque, NM 87106, USA; cabbott@salud.unm.edu (C.C.A.); dquinn@salud.unm.edu (D.K.Q.)

**Keywords:** rTMS, iTBS, neurostimulation, NIBS, hemispheric differences, MDD, treatment

## Abstract

Hemispheric differences in emotional processing have been observed for over half a century, leading to multiple theories classifying differing roles for the right and left hemisphere in emotional processing. Conventional acceptance of these theories has had lasting clinical implications for the treatment of mood disorders. The theory that the left hemisphere is broadly associated with positively valenced emotions, while the right hemisphere is broadly associated with negatively valenced emotions, drove the initial application of repetitive transcranial magnetic stimulation (rTMS) for the treatment of major depressive disorder (MDD). Subsequent rTMS research has led to improved response rates while adhering to the same initial paradigm of administering excitatory rTMS to the left prefrontal cortex (PFC) and inhibitory rTMS to the right PFC. However, accumulating evidence points to greater similarities in emotional regulation between the hemispheres than previously theorized, with potential implications for how rTMS for MDD may be delivered and optimized in the near future. This review will catalog the range of measurement modalities that have been used to explore and describe hemispheric differences, and highlight evidence that updates and advances knowledge of TMS targeting and parameter selection. Future directions for research are proposed that may advance precision medicine and improve efficacy of TMS for MDD.

## 1. Introduction

The implications of hemispheric laterality have been of interest to neuroscientists and the lay public alike for over half a century [1]. During that time, research has identified hemispheric differences with respect to cognitive function [2], biological sex [3], age group [4], and importantly, emotional processing [5,6,7]. More recently, the conceptualization of hemispheric emotional processing differences has informed the treatment of mood disorders and the application of noninvasive brain stimulation through techniques such as repetitive transcranial magnetic stimulation (rTMS). Initial rTMS studies for MDD found beneficial group effects when applying excitatory high-frequency rTMS (HF-rTMS) to the left dorsolateral prefrontal cortex (DLPFC) [8], and inhibitory low-frequency rTMS (LF-rTMS) to the right DLPFC [9]. This paradigm quickly became standard clinical convention [10], a status it has retained to date [11,12].

Despite numerous studies confirming that the left DLPFC is an efficacious target for HF-rTMS at the group level, response rates with these protocols remain between 25% and 45% [11,12,13], and a recent large controlled trial with negative results have led to questions about the generalizability of this approach [14]. Precision medicine strategies have demonstrated the potential to improve response rates when delivered to the left DLPFC [15]. These new strategies have been facilitated by advances in neuronavigation and individualized targeting [16,17], and by advances in rTMS application, specifically the advent of the intermittent theta burst stimulation (iTBS) paradigm [18], capable of greatly reducing the required treatment time and increasing the viability of clinical application [19]. 

Despite these advances, one-third to one-half of patients still do not respond to rTMS [20]. Given that patients who receive rTMS have often failed “first line” treatments for MDD like psychopharmacology [21], a response rate of 50% following rTMS among this population should not be disregarded, but the exploration of novel rTMS protocols might allow for even further improvement. While a selective review of studies beyond the rTMS literature provides ample support for the conventional hemispheric paradigm [22], in consideration of the ongoing problem of treatment resistance in major depressive disorder (MDD) it is reasonable to ask whether treatment delivered outside the typical paradigm may benefit patients who do not response to rTMS. Evidence for this possibility exists, as the neuroscientific literature is more equivocal than might be expected based on the focus of rTMS and MDD research to date, with theoretical and experimental work providing a rationale for reconsideration of early protocols and exploration of novel protocols, particularly in the right hemisphere.

The purpose of this scoping narrative review is to assess the prevailing theories of hemispheric differences in emotion processing across studies examining lesion location, electroencephalography, split-brain function, and structural and functional neuroimaging. Original search terms included, hemispheric asymmetry, emotional lateralization, hemispheric specialization, valence hypothesis. Additional search terms and relevant citations were gleaned from theoretical reviews of hemispheric differences in emotion processing [5,7,23], and reviews of rTMS in MDD and MDD neuroscience. Considerations for rTMS treatment research that incorporate an updated conceptualization of hemispheric similarities and differences for MDD treatment are proposed. 

## 2. Specialization vs. Valence

Two main theories have been proposed for right and left hemispheric differences in emotion processing. The first theory, hemispheric specialization, posits emotional processing as occurring predominantly in the right hemisphere, both for positive and negative emotions. A variant of this theory states that all initial emotional processing happens in the right hemisphere before being transferred to the left hemisphere for higher order appraisal and control [24,25,26]. The second theory posits the hemispheres as having divergent roles that depend upon the valence of a given emotion, where the right hemisphere is the processor of negative emotions and the left of positive emotions. A variant of this theory replaces positive and negative valence with approach (left hemisphere) and withdrawal (right hemisphere), making it consistent with findings associating anger generation with the left hemisphere [27,28,29], and anxiety generation in the right hemisphere [30,31], though both may be represented in diverse regions across the brain [32,33]. 

The field of noninvasive brain stimulation has tracked in line with the latter theory, following the observation that HF- and LF-rTMS are able to exert opposite effects on cortical excitability and neuronal metabolism [34,35,36]. Extensive work by Mayberg and others [37,38] demonstrating that hypometabolism/hypoactivity in the left frontal cortex was associated with depression provided the rationale for the first applications of HF-rTMS to the left DLPFC [8,39,40] and LF-rTMS to the right DLPFC [9,41,42]. Pascual-Leone’s 1996 study was the first evidence that the right hemisphere might respond oppositely to HF-rTMS compared to the left [8], setting the stage for numerous efficacy studies validating the left/excitatory, right/inhibitory paradigm [43].

### 2.1. Lesion Studies

Lesion studies form a relevant evidential base for understanding hemispheric differences as they pertain to rTMS application in MDD, as both single-pulse and repetitive TMS have been conceptualized as methods for generating virtual, temporary lesions [44,45]. Temporary and broad lesion-like effects were induced following the injection of sodium amobarbital as part of epilepsy surgery preoperative planning to determine hemispheric dominance for language [46]. Injection into the left carotid artery leading to temporary inactivation of the left hemisphere prompted reports of depressed mood, while injection into the right carotid artery with inactivation of the right hemisphere prompted reports of euphoria [47,48,49]. These findings were in line with emotional changes seen with lesions following stroke, where a higher propensity for depression was observed following strokes that damaged areas of the anterior left hemisphere. In contrast, damage to the anterior right hemisphere was observed to lead to an elevated mood state [50,51,52]. A similar pattern was seen among multiple sclerosis patients, with those with MDD displaying greater white matter damage to the left PFC [53]. These emotional changes following lesioning are thought to not only reflect the direct effect of the lesion itself, but also to represent a “release” of the unaffected side through loss of transcallosal inhibitory effects from the lesioned hemisphere [54,55].

In recent years, however, reviews have found mixed evidence in support of the connection between lesion location and depression [56], with some finding that right hemisphere lesions, rather than left, were more often associated with depression several months following stroke [57]. This was echoed in a recent meta-analysis that also found differences in the likelihood of depression in different periods following stroke damage, where right hemisphere lesions were associated with depression during the subacute phase immediately following a stroke, but after 6 months this association no longer held [58]. In contrast, another recent review found that lesions in the left hemisphere were more often associated with depression in the acute phase (1–3 months) following stroke, and that females displayed a greater likelihood of reporting depression with left hemisphere lesions [59]. Moderating factors such as sex are often at play in hemispheric differences between emotional processing and the manifestation of depression. Furthermore, consistent with a shifting conceptualization of MDD as a disorder of brain networks rather than brain regions [60,61,62,63], recent evidence suggests that the hemispheric location is less important than the alterations in the functional connectivity of large-scale networks that accompany lesions in specific cortical regions on either side [54].

### 2.2. Interoperative Brain Stimulation

For those receiving brain surgery for tumor removal, interoperative mapping of the brain through direct electrical stimulation is vital for maintaining critical functions following recovery. This includes the mapping of emotional appraisal [64]. Similarly, the implanting of intracranial electrodes is used in patients with intractable epilepsy to determine the epicenter of seizure activity prior to surgical intervention [65]. As a result of these interventions, researchers have been able to directly study brain regions and fiber tracts vital for emotional appraisal. Research from this area offers compelling evidence that the assessment of the emotional experience of others is a function of the right hemisphere [66,67]. In particular, the identification of emotional facial expressions in others involves cortical structures in the right hemisphere [68,69], a process that is distinct from the identification of faces generally [70]. Yet, while it is likely that emotional appraisal is centered in the right hemisphere, the experience of emotion itself is likely more diffuse. In an interoperative surgery case study, stimulating the cingulum bundle medial and inferior to the right superior temporal gyrus caused smiling and laughter, but in the absence of any underlying mirth [71]. In contrast, direct stimulation of the left anterior cingulum bundle elicited both smiling and laughter in additional to a positive emotional experience [72]. While it is likely that the exact location for eliciting mirth has heterogeneity across individuals [73], these targets are approaching those that have been suggested for deep brain stimulation for MDD [74], as well as recently proposed anticorrelated targets for rTMS [75].

### 2.3. Perceptual/Split-Brain Studies

Hemispheric differences in emotional processing have been explored in healthy adults through the dichotic listening and visual hemifield paradigms, together known as perceptual asymmetry. Dichotic listening tests involve simultaneous presentation of different auditory stimuli to both the left and right ear. A left ear/right hemisphere advantage is typically shown for the processing of emotional and nonverbal stimuli, while a right ear/left hemisphere advantage is shown for non-emotional and verbal stimuli [76]. Patients with mood disorders show evidence of hemispheric dysfunction in the dichotic listening paradigm, though the direction of this dysfunction varies, with those with anxiety disorders displaying worse performance for verbal stimuli (evidence for an underperforming left hemisphere), and those with MDD displaying worse performance for non-verbal stimuli (evidence for an underperforming right hemisphere) [77,78,79]. A similar pattern is seen in studies presenting lateralized visual stimuli, with those diagnosed with MDD having a reduced left hemi-spatial bias, and those with anxiety an increased bias [80]. Additionally, perceptual asymmetry differences are seen among subtypes of MDD, where a right hemispheric bias is observed in those with atypical MDD and dysthymia but not in those with MDD and melancholia [81]. Sex is also a moderating factor in perceptual asymmetry, with those with MDD demonstrating a reduced right hemisphere advantage for auditory stimuli that is more apparent among males [23].

Studies in patients who have had the corpus callosum severed, so-called “split-brain” patients, are complementary to those seen in perceptual asymmetry. The case of patient VP, as studied by Gazzaniga and Le Doux, provided an account of the emotional processing each hemisphere was uniquely capable of when VP was presented with stimuli to only the left or right hemisphere. When a frightening scene was presented to the right hemisphere’s visual field, VP was able to correctly identify the cause of the ensuing physiological arousal. However, when such a scene was presented to the left hemisphere, VP ascribed it to the unnerving nature of the room rather than the presented stimuli, suggesting that VP’s verbal left hemisphere was unable to accurately identify the cause of physiological change in the absence of communication from the right hemisphere [82]. Split-brain patients have also demonstrated poor recognition of emotional facial expressions presented to the left hemisphere, but good recognition when presented to the right [83], with the same relationship evident when interpreting emotionally laden written material [84] and when verbalizing emotion [85,86]. One interpretation of split brain studies is that the left hemisphere is able to make an interpretive best guess, but lacks the emotional comprehension provided by the right hemisphere [87]. Thus, split brain findings seem to support the idea that the majority of emotional appraisal, both positive and negative, occurs in the right hemisphere, rather than being distributed across the hemispheres according to valence or approach [5,88].

### 2.4. Electroencephalography Studies

In electroencephalography (EEG) studies, the relationship between the left and right PFC has been measured with frontal alpha asymmetry (FAA). According with behavioral results for perceptual asymmetry studies, FAA differences have been detected across patients with different mood disorder symptom profiles, with reduced activity observed over the left PFC in those diagnosed with MDD [30,89], and anxious apprehension associated with reduced activity in the right PFC compared to those with anhedonia [90]. This potential ability to identify subtypes of affective disorder as well as an ability to diagnose MDD led to efforts to use FAA as a diagnostic tool [91]. However, a recent meta-analysis found FAA had no diagnostic utility, at least in younger and middle aged adults [92]. In older adults (>53 years old) an interaction was observed in females diagnosed with MDD who presented with right-sided FAA, indicating greater cortical functionality in the left PFC compared to the right, while older males with MDD displayed left-sided FAA. In order to identify further actionable differences, it is likely that higher density EEG recording montages are needed, as treating the right and left PFC as functional units has often been due to measurement constraint rather than theoretical approach [6,93]. Even this may not prove sufficient, as FAA was not detected in a sample of 1008 MDD patients [94], despite performance of additional aggregative analyses [95].

### 2.5. Volumetric Studies

Examinations of brain volume changes reveal hemispheric differences related to mood disorder symptom profiles. Van Tol and colleagues observed that those with MDD unaccompanied by comorbid anxiety disorders had reduced brain volume in the right ventrolateral PFC (VLPFC) [96], a result that was subsequently supported in a meta-analysis of grey matter changes in MDD, where reductions across the right PFC were observed in MDD patients without comorbid anxiety [97]. However, the finding of reduced right VLPFC [98] and bilateral inferior frontal gyrus (IFG) [99] grey matter in those with anxious MDD has also been seen. Grey matter changes in the right superior frontal gyrus have also been shown to correlate with antidepressant medication treatment outcome, where treatment responders had higher, and treatment non-responders lower, grey matter volumes compared to controls [100]. Increases in grey matter volume have been seen following rTMS application for MDD, with subsequent symptom improvement correlated with volume increases in the anterior cingulate [101,102], a brain region implicated in multiple cognitive and emotional systems [103]. However, increases in cingulate cortex volume following rTMS coupled with an absence of clinical effects has also been observed [104], indicating that neuroplasticity changes alone may not be sufficient for symptom improvement, or that neuroplasticity changes may precede symptom improvement. Future research is needed, but grey matter volume may be a promising modality for measuring MDD subtypes and the effectiveness of rTMS interventions [105].

### 2.6. Molecular and Nuclear Imaging Studies

Early positron emission tomography (PET) studies found impaired left PFC metabolism in those with MDD [106], and improvements in this area were associated with positive treatment outcomes [107,108]. In the right PFC, both hypo- and hyperactivity have been demonstrated in patients with MDD [109,110]. In an early attempt at individualized precision medicine, Herwig and colleagues applied HF-rTMS to either the right or left DLPFC with the hemisphere of application determined by the side with more prominent hypometabolism as measured by PET. In most patients, the right hemisphere was identified as more hypometabolic. While a 30% reduction was seen in those who received left and right DLPFC rTMS, three patients receiving left DLFPC stimulation saw a 50% improvement in symptoms, compared to only one with right DLPFC stimulation [111] and overall the study did not improve on typical response rates [112]. A subsequent study used a similar design, with PET hypometabolism guiding HF-rTMS placement in 30 patients with left-sided hypometabolism and 16 with right-sided. Left sided stimulation was again superior at the group level, and in 7 of 16 receiving right-sided stimulation, only two displayed greater than 50% improvement in depression scores [113]. Based on these results, a generic approach would call for left-sided stimulation, but this runs the risk of ignoring a significant proportion of patients who benefit from right-sided HF-rTMS, possibly due to specific symptom profiles. In a single photon emission computed tomography study measuring blood flow rather than neuronal metabolism, those with melancholic MDD displayed a decrease in blood flow to both the left and right frontal lobes, while an increase in blood flow to the right frontal lobe was seen in patients with atypical MDD featuring symptoms such as increased appetite, sleep, and weight gain [114], highlighting the ongoing importance of determining how clinical profiles may influence rTMS efficacy [60].

### 2.7. White Matter Studies

Disruptions in white matter integrity have been observed in those with MDD, with white matter disruptions in the right hemisphere more widespread than those in the left [115]. In a recent study applying machine learning to diffusion tensor imaging (DTI) in patients with MDD and controls, a model containing fractional anisotropy maps of the right hemisphere alone was most successful in separating patients from controls, identifying those with MDD with 80% accuracy [116]. Other studies have implicated DTI findings in the corpus callosum, indicating that MDD may involve impaired hemispheric communication [116,117], though it is unclear whether this impairment is functional, structural, or both [118,119,120]. Adding further complexity to this picture, a study combining resting state functional connectivity MRI (rsfMRI) and DTI found that changes in the functional and structural coupling of intra-hemispheric communication correlated with depression severity, where greater depression severity was associated with greater functional-structural coupling [120]. Importantly, rTMS has demonstrated an ability to affect white matter tracts within the stimulated hemisphere [121,122]. Recent studies have demonstrated hemispheric differences in white matter topography underlying key cortical areas involved in mood and anxiety regulation [123,124,125], further suggesting that differences in structural connectivity between the left and right hemispheres in MDD likely have bearing on the efficacy of rTMS. This may be especially true in older adults, where it has been proposed that MDD is the result of age related degradation of white matter tracts [126].

### 2.8. Task-Related fMRI

Several task-related functional magnetic resonance imaging (fMRI) studies have provided support for a version of the valence model, indicating a hypoactive left PFC and a hyperactive right PFC during an emotion induction task in those diagnosed with MDD [127,128,129]. In contrast, other studies have found that depressed patients demonstrate a hypoactive right PFC compared to controls during emotion induction [128,130,131], coupled with left PFC hyperactivity [127]. This heterogeneity is summarized in a meta-analysis of task-based activation studies where no brain regions emerged as significantly different in those with MDD compared to controls across 99 neuroimaging experiments [132]. In addition to experimental differences in stimuli and analysis, differences in age, sex, medication status, and MDD subtypes likely also lead to contrasting results. In one interesting task-based study that accounted for subtypes of MDD, those with high anxious arousal and low anxious apprehension demonstrated increased activity in the right DLPFC and reduced activity in the right VLPFC [133]. This difference across types of symptom profiles may help explain contrasting results seen in other measurement modalities, such as in grey matter and PET changes in the right PFC, and points toward emerging work suggesting that variable activity at specific depression network nodes is likely to underlie different symptoms [60].

### 2.9. Functional Connectivity fMRI Studies

Of the imaging modalities included in this review, rsfMRI has played the largest role in advancing precision medicine applications of rTMS in MDD over the last decade. Building on evidence indicating that alterations in the subgenual anterior cingulate cortex (sgACC) provide a meaningful biomarker in MDD [134,135], Fox and colleagues assessed whether rTMS efficacy could be predicted by examining functional connectivity between the left DLFPC and sgACC. They found that DLPFC stimulation sites with greater anticorrelation with the sgACC were more effective targets, with the degree of anticorrelation accounting for 70% of the variance in treatment outcome [136]. The viability of this method for improving personalized targeting has since been replicated, improving treatment response rates to between 44% and 90% [137,138,139,140], a significant improvement over response rates seen in earlier treatment studies of rTMS for MDD. 

Despite this improvement, it is likely that a significant proportion of non-responders to left DLPFC stimulation may still benefit from rTMS, as indicated by the PET studies which found that a small number of subjects responded dramatically to HF-rTMS delivered to the right PFC [111,113]. Such a possibility fits with the finding that over 1000 unique symptom profiles are possible in MDD [141]. Work identifying the HF-rTMS right PFC symptom profile has begun [142], and emerging evidence suggests that, similar to findings with other experimental paradigms, comorbid anxiety alters resting state functional connectivity (rsFC) to the right PFC, with altered connectivity between right VLPFC and limbic system in patients with comorbid anxiety compared to those with MDD only [143]. In an intriguing directed functional connectivity analysis, a subgroup with anxiety, recurrent MDD and greater female representation displayed effective connectivity emanating from the VLPFC to the right parietal lobe [144]. Similarly, patients divided into anxiosomatic and dysphoric depression subtypes had contrasting PFC targets, with anxiosomatic symptoms responding more avidly to posteromedial rTMS treatment targets, and dysphoric patients having targets in both hemispheres more anterolateral to targets derived from traditional DLPFC targeting [145]. In a large study using data from over 1000 subjects, MDD subtypes were delineated based on rsFC [60] with a single biotype loading on fatigue and anergia most strongly associated with the right ventrolateral PFC with concurrent reduced connectivity to the anterior cingulate. In an important test of their approach, HF-rTMS targeting the DMPFC of patients in each of four identified biotypes resulted in significantly contrasting response rates. Those classified as biotype 1, with a symptom profile of fatigue/anergia, middle insomnia, and anxiety responded at a rate of 82.5% (greater than 25% reduction in depression scores) compared to a response rate of 61%, 25% and 29.6% for the other three biotypes. Given these findings of specific symptoms responding better to specific placements, we expect that the connectivity phenotypes observed in rsFC studies such as by Drysdale et al. may indicate a differential responsivity to stimulation across targets, such as the DLPFC or DMPFC.

Finally, Siddiqi et al. in 2021 published a cross-cutting study of combined rsfMRI data from eight heterogeneous studies examining connectivity changes associated with post-stroke MDD, rTMS for MDD, and deep brain stimulation for MDD [63]. A common depression network was revealed that partially overlaps with the executive and salience networks, with correlation between nodes in the inferior frontal gyrus (IFG), DLPFC, dorsal ACC, and posterior parietal cortex (PPC), and anticorrelation with the default mode network and nodes in the subgenual cingulate cortex and ventromedial prefrontal cortex. While this study lends support to the approaches of targeting rTMS that have succeeded thus far, namely, to target the DMPFC, the DLPFC, and even the IFG, what is noteworthy in this study from a laterality perspective is the symmetry of network topography between the left and right hemispheres, which argues against a lateralization of emotional regulation. If the important treatment targets for rTMS are bilaterally distributed network nodes, then the location of stimulation should be determined by which nodes offer the most efficacious ingress point for influencing important subcortical structures such as the amygdala [146], PCC [125], and sgACC. It is possible that indirect stimulation of the sgACC may be best achieved through nodes in the right hemisphere, which have potentially more robust connections through the anterior insula to the sgACC and function critically as part of the central executive and salience networks [147,148,149]. Please refer to Table 1 for a brief summary of reviewed findings. 

## 3. Implications for rTMS for MDD

While this paper began by reviewing theories of hemispheric emotional processing differences, it is possible that thinking in terms of hemispheric differences represents a binary fallacy, more representative of how humans think than of the actual structure and function of the human brain. Hemispheric lateralization in various domains of cognition, emotional processing, personality, and behavior selection has become a common allusion in mainstream Western society (e.g., “left-brain” versus “right-brain” people), and clinical researchers are not immune to the allure of an elegant binary formulation, particular if it is associated with clinical treatment paradigms. Influential findings in early lesion studies and split-brain studies provided the initial empirical bases for theorized differences in hemispheric function, with follow-up studies using sophisticated imaging techniques such as EEG, structural and functional MRI, and PET describing a more complex and qualified picture of hemispheric brain function, particularly through the valence theory of emotional processing. The behavioral effects in multiple studies of rTMS for MDD based on these principles of hemispheric lateralization have largely conformed to expectations, with far fewer studies having been conducted to provide counter evidence to this theory (e.g., right/excitatory, left/inhibitory). 

However, evidence against the “restoration” of a beneficial or optimal balance between the left and right hemispheres concurrent with the alleviation of MDD through the left/excitatory, right/inhibitory stimulation paradigm is starting to emerge. First, imaging studies of rTMS effects in MDD demonstrate changes in network interactions that can span both hemispheres [150], and through recent rsfMRI studies a bilateral depression network appears to be coalescing with symmetric nodes in the left and right hemispheres [63]. Dynamics between these bilateral networks, such as the executive, salience, and default mode networks, appear to be more relevant than the dynamics between left and right hemisphere, both for MDD [151] and psychopathology broadly [62]. Second, excitatory stimulation patterns to the right hemisphere, such as 10Hz HF-rTMS and intermittent theta burst stimulation (iTBS) can also have antidepressant effects [142,152,153,154], indicating that contrary earlier findings may not have undergone sufficiently rigorous confirmation. Through the study of stimulation paradigms symmetrically across both hemispheres, it will be possible to understand how the hemispheres innately function in emotion processing and mood regulation. For instance, it is widely believed that anxiety may be a symptom emanating more from right hemisphere activity, and that treatment directed at the right hemisphere can have anxiolytic effects [155,156,157]. With excitatory patterns being applied to the right hemisphere, it will be possible to quantify to what degree anxiogenesis or anxiolysis may occur in comparison to the left hemisphere. Third, clinical anecdote and naturalistic studies indicate that hemispheric lateralization may occur more at the level of the patient rather than at the population level, with specific individuals having variable levels of depression network plasticity in a given hemisphere, suggesting that antidepressant brain stimulation for a given patient will need to be directed to either the left, right, or both hemispheres based on their specific network activity.

The increasing use of symmetric protocols (where the same parameters are applied to both sides of the brain) will allow for an understanding of these differences. This is not to suggest that the field should abandon inhibitory protocols, as many patients have seen successful remission of MDD with 1 Hz stimulation; rather, we suggest that the personalization of rTMS will need to include laterality as one of at least four levels of personalization: (1) identification of target locations through functional connectivity studies, such as the sgACC-DLPFC anticorrelated target; (2) identification and characterization of which anticorrelated network nodes are the optimal targets, such as the IFG, dorsal ACC, and PPC; (3) identification of how strongly the laterality of targets factors into clinical efficacy; (4) and identification of how stimulation parameters themselves vary in their effects depending on target location and laterality. 

Improving the efficacy of MDD treatment will also likely entail the formulation of theories that are able to integrate various phenotypes seen in MDD subpopulations. Demographic and clinical features such as sex, age, and the presence of comorbid psychiatric disorders will undoubtedly play a role in guiding future research and determining optimal personalized treatment that accounts for comorbidities. A theoretical understanding of changes common to specific populations with lower response rates, such as interhemispheric reorganization of brain activity seen in aging [4,158], can help to guide subsequent individualized approaches within those populations. Recognition that the alluring binary of right versus left hemispheric differences in emotional processing is at best simplistic and at worst iatrogenic is a first step towards improving theoretical understanding and treatment outcomes following rTMS MDD treatment. 

## Figures and Tables

**Table 1:** Brief summary of findings.

Paradigm	Difference	Effect
Sodium amobarbital injection	Injection into the left versus right carotid artery	Inactivation of the left hemisphere led to temporary depression, while inactivation of the right hemisphere led to temporary euphoria [47,48,49]
Lesion Studies	Lesions occurring in the anterior right versus left hemisphere	A higher likelihood of depression observed following damage to the left hemisphere in contrast to an elevated mood following damage to the right hemisphere [50,51,52]
Lesion Studies	Time following stroke	Lesioning of the right hemisphere only associated with depression in the months following stroke [57,58]
Lesion Studies	Individual moderating factors	Whether or not a lesion location is associated with depression dependent upon moderating factors like sex [59]
Lesion Studies	Functional connectivity of lesion location	Functional connectivity changes that accompany lesions are more important for depression than lesion location in either hemisphere [54]
Interoperative Brain Stimulation	Emotional processing versus affective experience	Right hemisphere strongly associated with identification of emotions in others [69,70]
Dichotic Listening	Advantages in processing auditory stimuli associated with the right or left hemisphere	Those with depression have worse performance processing non-verbal stimuli, while those with anxiety have worse performance processing verbal stimuli [77,78,79]
Perceptual Asymmetry	Hemi-spatial bias to visual stimuli presented to the right or left hemisphere	A right hemisphere bias is seen in those with depression and dysthymia but not in those with depression and melancholia [81]
Split Brain Patients	Presentation to the right or left hemisphere visual field only	Attributions for emotional changes brought on by viewing emotionally salient images only correct when seen through right hemisphere visual field [82]
Frontal Alpha Asymmetry	EEG activity in right and left frontal lobes	Reduced activity in the left PFC seen in those diagnosed with MDD, compared to reduced activity in the right PFC in those with anxious apprehension [30,89,90]
Frontal Alpha Asymmetry	EEG activity in right and left frontal lobes in older adults diagnosed with MDD	In females over the age of 53 diagnosed with MDD, hyperactivity of the left PFC was associated with depression, compared to hyperactivity of the right PFC in men over 53 [92]
Volumetric Studies	PFC brain volume in those with MDD with or without comorbid anxiety	Those with MDD only had reduced brain volume in the right PFC; but the opposite finding has also been observed [96,97,99]
Volumetric Studies	Brain volume changes following antidepressant medication treatment	Responders to treatment had increases in grey matter compared to controls while non responders had decreases [100]
Volumetric Studies	Brain volume changes following rTMS treatment	Increases in anterior cingulate cortex volume following rTMS associated with symptom improvement, though increases in volume also observed in the absence of clinical benefit [101,102,104]
Positron Emission Tomography	Differences in metabolism in the left PFC	Impaired metabolism observed in the left PFC in those with MDD and improvements in metabolism associated with positive treatment outcomes [106,107,108]
Positron Emission Tomography	Differences in metabolism in the right PFC	Both hypo and hyper activity seen in those with MDD [109,110]
Positron Emission Tomography	Differences in PFC metabolism guiding rTMS placement	While the right PFC was more often selected for treatment based on hypometabolism, this method did not lead to improved outcomes [111,113]
White Matter Studies	Differences in white matter integrity between left and right hemispheres in those with MDD	Those with MDD have more widespread white matter disruptions in right hemisphere compared to left, right hemisphere differences alone successful in correctly identifying those with MDD with 80% accuracy [115,116]
White matter studies	Differences in white matter integrity in the corpus callosum in those with MDD versus controls	Those with MDD may have impaired interhemispheric communication [116,117]
Task Based fMRI	Differences in BOLD in the right and left PFC in those with MDD	Some studies have identified a pattern of hypoactivity in the left PFC and hyperactivity in the right in those with MDD during an emotion induction task, while others have identified a hypoactive right PFC in those with MDD in the same task [127,128,129,130,131]
Functional Connectivity	Individual moderating factors	Factors such as comorbid anxiety and sex may change connectivity patterns to bias right hemisphere treatment targets in rTMS [60,143,144]
Functional Connectivity	A matter of networks rather than hemispheres	Differences in cross hemispheric networks are more important in MDD than hemispheric location [63]
